# Solid fuel use, socioeconomic status and depression: a cross-study of older adults in China

**DOI:** 10.1186/s12877-024-04670-6

**Published:** 2024-01-30

**Authors:** Ying Duan, Zihao Liu, Qi Qi, Huaqing Liu, Min Zhang

**Affiliations:** 1 School of Public Health, Bengbu Medical University, Bengbu, Anhui China; 2 School of Health Management, Bengbu Medical University, Bengbu, Anhui China

**Keywords:** Solid fuel, Socioeconomic, Depression, Older adults

## Abstract

**Background:**

Indoor air pollution causes severe psychological stress and promotes depression. A better understanding of the impact of solid fuel consumption and socioeconomic indicators on mental health is critical to promote successful aging. In this study, we analyzed the relationship of depression with socioeconomic status (SES) and solid fuel use, and illustrated the mediating role of solid fuel use in the relationship between SES and depression.

**Methods:**

9250 participants from the 2018 wave of the Chinese Longitudinal Healthy Longevity Survey were included in this study. A logistic regression analysis was used to calculate odds ratio (OR) and 95% confidence interval (CI) of depression for different types of fuel consumption. The stepwise approach and the Sobel test were used to test the mediation effect.

**Results:**

Older people who reported the consumption of solid fuels showed higher odds of having depressive symptoms (OR = 1.16, 95% CI:1.03, 1.31). In model with depression as the outcome variable, the ORs of low education level and low annual household income level were 1.30 (95% CI: 1.15, 1.47) and 1.43 (95% CI: 1.28, 1.59) respectively. Solid fuel consumption accounted for 38.40% of the effect of a low education level and 54.73% of the effect of low income on depression.

**Conclusions:**

Solid fuel use and SES are associated with depression, and solid fuel use may act as a potential mediator connecting socioeconomic indicators and depression.

**Supplementary Information:**

The online version contains supplementary material available at 10.1186/s12877-024-04670-6.

## Introduction

Ambient air pollution is increasingly seen as a severe public health issue, particularly indoor air pollution in developing countries [1]. It ranks among the top five mortality risk factors in countries with developing economies such as China [2]. At present, the impact of outdoor air pollution on health is the focus of most studies [3]. However, indoor air pollution is also a vital part of ambient air pollution, particularly considering that people are at home for a long period of time every day. It was calculated that stoves fueled by coal and biofuels (wood, animal manure, droppings, crop waste, and charcoal) are used by 2.4 billion people [4]. For cooking and heating, rural Chinese people mostly rely on solid fuels, which account for 61% and 15% of total energy use, respectively [5]. Therefore, for the health of Chinese residents, indoor air pollution generated by solid fuel combustion is an inevitable hazard.

The aging of the global population is accelerating rapidly. Predictions indicate that in 2050, the number of people over 65 years of age will reach 400 million, and the number of people over 80 years of age will reach 150 million [6]. As one of the countries with the fastest aging population in the world, China had 0.191 billion elderly people aged 65 and above, accounting for 13.5% of China’s total population in 2020 [7]. A core issue in the process of aging is age-related health problems [8]. With increasing age, in addition to the rise in comorbidities, older adults are also considered to have the possibility of developing depression and anxiety [9]. Depression is a major public health problem affecting older people in China. One study showed that 20.3% of older individuals in China suffer from depression [10], which places a serious burden on families and society. Additionally, depression accompanies lower cognition, physical performance, and social capabilities [11].

There are four studies evaluating the link between depression and indoor solid fuel consumption [12–15]. Incomplete combustion of solid fuels leads to increased emissions of pollutants such as carbon monoxide and particulate matter (PM) [16], and these substances may increase the risk of mental disorders such as depression through cerebrovascular injury, oxidative stress, neuroinflammation or neurodegeneration [17–20]. SES is an important factor influencing indoor air pollution exposure [21], and people with low household incomes or low levels of education have higher indoor air pollution exposure [22]. Clean fuels are typically more expensive relative to solid fuels [23], so people with low SES may prefer cheaper options. SES is also associated with mental health vulnerability. In low SES groups, the combination of risk factors such as greater exposure to adversity, less social support and fewer resources to cope with stress can lead to more severe depressive outcomes [24–26]. On the one hand, solid fuel consumption is influenced by SES, and on the other hand, the prevalence of depression is associated with solid fuel consumption. However, the relationship among solid fuel consumption, SES, and depression remains unclear. Recent research [27] has reported on the mediating role of solid fuel consumption in cardiovascular disease risk associated with SES. Therefore, we speculated that there may be a similar association among solid fuel consumption, SES, and depression. This study estimated the association of depression with solid fuel use and SES, and the mediating role of solid fuel use in the relationship between SES and depression.

## Methods

### Study population

We employed data from the eighth wave (the most recent survey in 2018) of the Chinese Longitudinal Healthy Longevity Survey (CLHLS), which was conducted in approximately 630 counties/cities throughout 22 mainland Chinese provinces. Using a standardized questionnaire, trained investigators gathered data on demographic factors, lifestyle behaviors, and health status. Face-to-face interviews were performed at the participants’ homes. Investigators assisted illiterate individuals in completing the questionnaire. More detailed information about the CLHLS is available elsewhere [28].

**Fig. 1:**
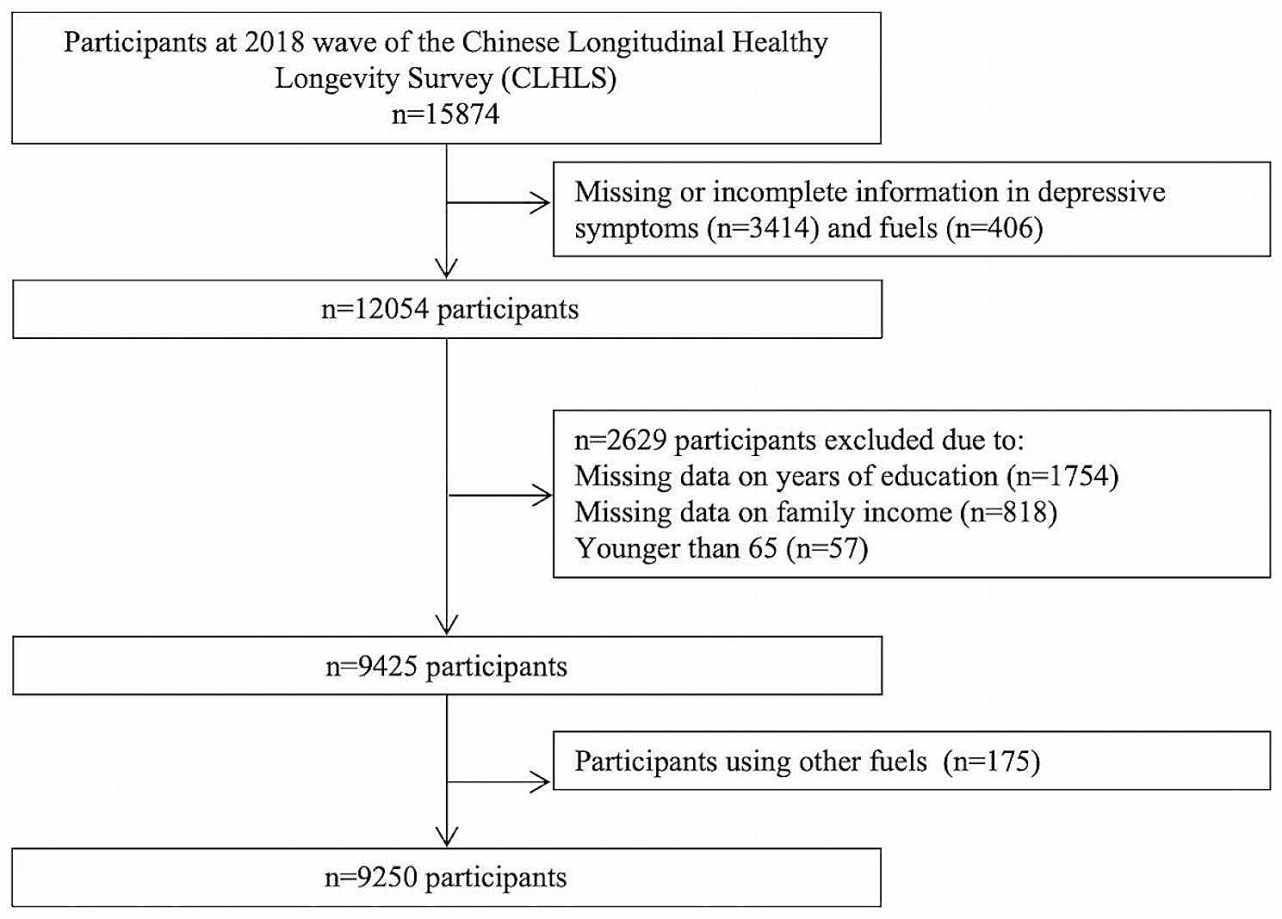
Flow chart of the selection of study participants

In the survey, 15,874 participants were interviewed. For our analysis, 6449 participants were excluded because of missing data on depression (3414), cooking fuels (406), years of schooling (1754), annual household income (818), and age < 65 years (57). Participants classified as “others” were omitted from the cooking fuel subgroup of the study to avoid obscuring the results. Finally, a total of 9250 older adults were included in the study. Figure 1 depicts the research participant inclusion and exclusion process in this investigation. The missing participants were more likely to be female; aged 80 years or above; live in rural areas; be divorced or widowed; not smoke, drink, play cards, participate in social activities or travel; eat fruits and vegetables more often; have a normal body mass index (BMI); and not have hypertension, diabetes, heart disease, or stroke.

### Definition of primary variables

The type of fuel consumed was measured by using one question: “Which fuels are commonly used for cooking in your home?” The responses included solid fuel (e.g., charcoal, firewood, and straw), clean fuel (e.g., electricity, gas and solar energy), never cooking at home and other types of fuel. Those who reported never cooking or reported using other types of fuel at home were categorized as “others”.

Income and education level were the two main socioeconomic indicators considered in our research. The CLHLS collected the specific years of schooling and annual household income of each participant. To simplify the mediation analysis, we classified education level into “no school” and “1 year or more”. According to the median of all samples, the annual household income (yuan) was divided into “≤30 000” and “>30 000”.

In this investigation, depressive symptoms were assessed with the 10-item Center for Epidemiologic Studies Depression Scale (CES-D-10) [29]. The responses were categorized into four categories, “rarely”, “some days”, “sometimes”, and “most of the time”, and correspondingly coded as 0, 1, 2 and 3. However, the responses to two positive questions, i.e., “I was glad” and “I felt hopeful about the future”, were reverse coded. The CES-D-10 total score ranges from 0 to 30, and the higher the score is, the more serious the depression. If an individual receives a score of more than 10, he or she is deemed to have depressive symptoms. This 10-point cutoff has been frequently utilized in earlier studies [14, 30] and has been well validated in the evaluation of depression in Chinese older adults, independent of age or dementia status [31–32].

### Definitions of other variables

Based on previous studies [33–35], demographic variables, lifestyle behaviors, and health status may be potential confounding factors, and we included them as covariates in the analysis. Demographic variables included age, sex (“men” and “women”), residence status (“urban area” and “rural area”), and marital status (“married”, “unmarried”, “divorced/widowed”). Lifestyle behaviors included smoking status, drinking status, playing cards status, participation in social activities (“yes” and “no”), ventilation status (according to participants’ responses to the question “Ventilation of the kitchen when cooking at home”, ventilation situation was defined as “no” for participants who did not take ventilation measures, “yes” for participants who took ventilation with hoods, exhaust fans and open windows, and “unknown” for those who did not know about ventilation), tourism status (classified into “0” or “≥1 time” according to a participant’s response to the question “How many trips outside of your home city/county have you made in the past two years?”), exercise status (“yes” and “no”) and the consumption of fresh fruits and vegetables (“almost or quite often”, “occasionally” and “rarely or never”). The health status included BMI (kg/m^2^) and self-reported history of diseases, which included hypertension, diabetes, heart diseases, and stroke (“yes”, “no” and “unknown”). According to their BMI, participants were defined as “underweight (< 18.5 kg/m^2^)”, “normal (18–23.9 kg/m^2^)”, “overweight (24–27.9 kg/m^2^)”, and “obese (≥ 28 kg/m^2^)”.

### Statistical analysis

Statistical analysis was conducted with SPSS 17.0 and Stata v16.0. Categorical data were described using numbers and percentages, continuous data were described using mean and standard deviation (SD), and chi-square test or two-sample *t* test were employed to verify their differences in fuel consumption across participant characteristics, respectively. A logistic regression analysis was utilized to estimate the odds ratios (ORs) of depressive symptoms for solid fuel consumption (clean fuel as the reference group). Effects of education and income levels on depression separately after adjusted age, sex, body mass index, smoking status, marital status, alcohol status, residence status, tourism status, exercise status, playing cards status, participation in social activities, ventilation status, consumption of fresh fruit and vegetables, and self-reported history of diseases.

We assumed that some variables were the true reason for the elevated risks of depression among solid fuel consumers. Solid fuel consumption was another consequence of this real cause and thus served as a “bridge” in the crude model. Hence, we constructed a causal relationship using two possible SES parameters (annual household income and education level). The overall effect of SES on depression, the influence of SES on solid fuel usage, and the effect of solid fuel consumption on depression were all estimated using logistic regression in the mediation analysis. The stepwise technique [36], of which more details are shown in the Supplementary file (the second part), was employed to determine whether solid fuel consumption acts as a mediator between socioeconomic position and depression. In addition, the Sobel test [37] was used to ensure that we did not overlook any potential mediator effects and to give us additional confidence in our findings. A Z-score was calculated to assess the mediation effect for categorical variables (Supplementary file, Eq. 4).

To assess the robustness of our results, we also performed some additional sensitivity analyses. A 12-point cutoff of the CES-D-10 has also been used to identify clinical depression in older Chinese people in some previous studies [38]. Therefore, we repeated all analyses with 12 as the cutoff value. Statistical significance was defined as a two-sided *P value* threshold of 0.05.

## Results

### Basic characteristics of the participants

Table 1 shows the participants’ characteristics in the 2018 wave of the CLHLS based on the type of fuel they utilized. The average age of participants is 82.9 years with SD of 11.4 years, of which 53.90% are women. A total of 71.80% of the older people consumed clean fuel, 28.20% consumed solid fuels. Among the participants who consumed clean fuels, 61.87% had one year of school experience or more, and 58.96% had an annual household income of more than 30 000 yuan. Among those who consumed solid fuels, 56.22% had no school experience, and 79.07% had an annual household income of 30 000 yuan or less.

**Table 1:** Characteristics of the study participants

Characteristics	n	Clean fuel	Biomass fuel	P value for difference
**Total**	9250	6637(71.8)	2613(28.2)	
**Age (mean ± SD)**	82.9 ± 11.4	82.9 ± 11.4	83.0 ± 11.3	0.617
**Sex**				
Men	4265(46.1)	3108(72.9)	1157(27.1)	0.027
Women	4985(53.9)	3529(70.8)	1456(29.2)	
**Education**				
No school	4000(43.2)	2531(63.3)	1469(36.7)	<0.001
1 year or more	5250(56.8)	4106(78.2)	1144(21.8)	
**Residence status**				
Urban area	1582(17.5)	1488(94.1)	94(5.9)	<0.001
Rural area	7479(82.5)	5014(67.0)	2465(33.0)	
**Annual household income (yuan)**				
≤ 30 000	4779(51.7)	2724(57.0)	2055(43.0)	<0.001
>30 000	4471(48.3)	3913(87.5)	558(12.5)	
**Marital status**				
Unmarried	59(0.6)	36(61.0)	23(39.0)	0.040
Married	4512(49.2)	3204(71.0)	1308(29.0)	
Divorced/ Widowed	4607(50.2)	3347(72.7)	1260(27.3)	
**Smoking status**				
Yes	1512(16.5)	1021(67.5)	491(32.5)	<0.001
No	7653(83.5)	5547(72.5)	2106(27.5)	
**Drinking status**				
Yes	1406(15.4)	969(68.9)	437(31.1)	0.009
No	7701(84.6)	5570(72.3)	2131(27.7)	
**Ventilation status**				
Yes	8450(91.4)	6235(73.8)	2215(26.2)	<0.001
No	783(8.5)	394(50.3)	389(49.7)	
unknown	16(0.2)	7(43.8)	9(56.3)	
**Participate in social activities**				
Yes	917(9.9)	794(86.6)	123(13.4)	<0.001
No	8333(90.1)	5843(70.1)	2490(29.9)	
**Play cards status**				
Yes	1459(15.8)	1130(77.5)	329(22.5)	<0.001
No	7791(84.2)	5507(70.7)	2284(29.3)	
**Exercise status**				
Yes	3257(35.7)	2647(81.3)	610(18.7)	<0.001
No	5879(64.3)	3903(66.4)	1976(33.6)	
**Tourism status**				
≥ 1 times	1400(15.1)	1227(87.6)	173(12.4)	<0.001
0	7850(84.9)	5410(68.9)	2440(31.1)	
**Fresh fruit**				
Almost or quite often	4434(47.9)	3532(79.7)	902(20.3)	<0.001
Occasionally	2697(29.2)	1781(66.0)	916(34.0)	
Rarely or never	2119(22.9)	1324(62.5)	795(37.5)	
**Vegetables**				
Almost or quite often	8380(90.6i)	6078(72.5)	2302(27.5)	<0.001
Occasionally	607(6.6)	381(62.8)	226(37.2)	
Rarely or never	2663(2.8)	178(67.7)	85(32.3)	
**Body mass index (kg/m2)**				
Underweight (< 18.5)	1483(16.1)	982(66.2)	501(33.8)	<0.001
Normal (18.5–23.9)	593(49.8)	3185(69.3)	1408(30.7)	
Overweight (24–27.9)	2304(25.0)	1779(77.2)	525(22.8)	
Obese (≥ 28)	844(9.2)	669(79.3)	175(20.7)	
**Hypertension**				
Yes	3991(43.2)	3057(76.6)	934(23.4)	<0.001
No	4643(50.2)	3154(67.9)	1489(32.1)	
Unknown	614(6.6)	425(69.2)	189(30.8)	
**Diabetes**				
Yes	998(10.8)	840(84.2)	158(15.8)	<0.001
No	7273(78.6)	5127(70.5)	2146(29.5)	
Unknown	977(10.6)	668(68.4)	309(31.6)	
**Heart diseases**				
Yes	1608(17.4)	1298(80.7)	310(19.3)	<0.001
No	6700(72.4)	4696(70.1)	2004(29.9)	
Unknown	940(10.2)	641(68.2)	299(31.8)	
**Stroke**				
Yes	980(10.6)	760(77.6)	220(22.4)	<0.001
No	7267(78.6)	5187(71.4)	2080(28.6)	
Unknown	1003(10.8)	690(68.8)	313(31.2)	

**Table 2:** Odds ratio (95% CI) of depression with solid fuel consumption

	Clean fuel	Solid fuel	P
**A CES-D-10(cut-off value = 10)**			
Model 1^a^	Reference	1.55(1.41,1.71)	<0.001
Model 2^b^	Reference	1.49(1.35,1.65)	<0.001
Model 3^c^	Reference	1.28(1.14,1.43)	<0.001
Model 4^d^	Reference	1.16(1.03,1.31)	0.013
**B CES-D-10(cut-off value = 12)**			
Model 1	Reference	1.55(1.38,1.75)	<0.001
Model 2	Reference	1.49(1.32,1.67)	<0.001
Model 3	Reference	1.29(1.13,1.48)	<0.001
Model 4	Reference	1.19(1.04,1.37)	0.014

CES-D, Center for Epidemiological Studies Depression Scale

^a^ Not adjusted

^b^ Adjusted for ventilation status

^c^ Adjusted for age, sex, body mass index, smoking status, marital status, alcohol status, residence status, tourism status, exercise status, playing cards status, participation in social activities, consumption of fresh fruit and vegetables, and self-reported history of diseases based on Model 2

^d^ Adjusted for annual household income and education level based on Model 3

### Associations of depression with solid fuel consumption

Older people who consumed solid fuels had a higher risk (OR = 1.55, 95% CI: 1.41, 1.71) of having depressive symptoms in the crude model (Table 2A), and this association still existed (OR = 1.16, 95% CI: 1.03, 1.31) after adjustment for covariables (age, sex, smoking status, drinking status, marital status, residence status, ventilation status, tourism status, exercise status, playing cards status, participation in social activities, consumption of fresh fruits and vegetables, BMI, self-reported history of diseases, household income and education level). We further conducted a sensitivity analysis using 12-point instead of 10-point as the cutoff for identifying depression (Table 2B), and this positive association did not change (OR = 1.55, 95% CI: 1.38, 1.75 in the crude model; OR = 1.19, 95% CI: 1.04, 1.37 in the final model).

### The mediating role of fuel consumption in the relationship between socioeconomic status and depression

Among those who cooked on a regular basis, there were considerable links between poor socioeconomic status and depression. In the final model with depression as the outcome variable, the ORs of low education level and low annual household income level were 1.30 (95% CI: 1.15, 1.47) and 1.43 (95% CI: 1.28, 1.59) respectively (Table 3). We discovered a robust link between the consumption of solid fuels and depression. As a result, solid fuel consumption acted as a mediator in the influence of SES on depression, considering the requirements of a stepwise procedure. Based on the Sobel test, we came to the same conclusion. In the model with depression as the outcome variable, the Z-statistics of education level and annual household income level were − 3.10 and − 2.34, respectively (both less than − 1.96). The mediating effect of solid fuel consumption accounted for 38.40% of the full effect of a low education level on depression and 54.73% of the full effect of poverty on depression (Table 4). The coefficients of all covariates in the mediation model are shown in Table S1 of the Supplementary file.

**Table 3:** Odds ratio (95% CI) of depression with socioeconomic status

	CES-D-10(cut-off value = 10)	CES-D-12(cut-off value = 12)
**Education**		
1 year or more	Reference	
No school ^a^	1.30(1.15, 1.47)	1.21(1.05, 1.40)
**Annual Household Income**		
Above 30,000 yuan/year	Reference	
Below 30,000 yuan/year ^a^	1.43(1.28, 1.59)	1.39(1.22, 1.59)

CES-D, Center for Epidemiological Studies Depression Scale

^a^Odds ratio and 95% CI. The model was adjusted for age, sex, body mass index, smoking status, marital status, alcohol status, residence status, tourism status, exercise status, playing cards status, participation in social activities, ventilation status, consumption of fresh fruit and vegetables, and self-reported history of diseases

**Table 4:** The mediating effect of solid fuel on the association between socioeconomic status and depression

	CES-D-10(cut-off value = 10)	CES-D-12(cut-off value = 12)
**Education**		
c	-0.25	-0.17
a	-0.48	-0.48
b	0.20	0.21
c’	-0.23	-0.15
Zab for Sobel-test	-3.10	-2.90
Mediation effect percentage	38.40%	59.29%
**Annual Household Income**		
c	-0.33	-0.31
a	-1.30	-1.30
b	0.14	0.16
c’	-0.29	-0.27
Zab for Sobel-test	-2.34	-2.24
Mediation effect percentage	54.73%	67.10%

CES-D, Center for Epidemiological Studies Depression Scale

c: regression coefficient of socioeconomic status on depression in Eq. 1 of the supplementary file

a: regression coefficient of socioeconomic status on solid fuel in Eq. 2 of the supplementary file

b: regression coefficients of solid fuel on depression in Eq. 3 of the supplementary file

c’: regression coefficients of socioeconomic status on depression in Eq. 3 of the supplementary file

Zab for Sobel-test was calculated according to Eq. 4 of the supplementary file

Mediation effect percentage was defined as the role of fuel type in the effect of socioeconomic status on depression and was calculated according to Eq. 5 of the supplementary file

When using 12 as the cutoff value, the mediating effect still existed. The model’s Z-statistics using depression as the outcome variable were − 2.90 and − 2.24, respectively. The mediating effects of low education and income levels on the total effect of depression were 59.29% and 67.10%, respectively (Table 4).

## Discussion

Solid fuels are the primary source of indoor air pollution. In economically deprived places, such as rural communities, the consumption of solid fuel can be more common. Harmful chemicals and particles released by solid fuel combustion affect our physical and mental health. It is crucial to investigate the links between household fuel consumption and depression. In keeping with the results of existing studies [12–15], we discovered that solid fuel consumers were more likely to be depressed than clean fuel consumers. And we found that low SES was linked to high solid fuel consumption, which in turn was linked to more symptoms of depression. Using the stepwise method and the Sobel test, we found that solid fuel consumption was a mediator of socioeconomic status and the incidence of depression.

Solid fuel combustion produces significantly higher levels of gas pollutants, which have linked to depression, than clean fuel combustion. A lack of dopamine in the central nervous system is associated with depression [39], and oxidative stress caused by air pollution leads to the death of dopamine neurons [18]. Higher PM exposure may induce metabolic alterations that are consistent with the activation of the hypothalamus-pituitary-adrenal axis, which stimulates the synthesis and release of cortisol [40]. Research shows that cortisol is related to the development of depression [41]. Animal studies have also shown that reduced plasma tryptophan levels are associated with air pollutants [42]. A decrease in tryptophan levels reduces serotonin synthesis [39]. Serotonin levels are inversely associated with depression risk [43].

The socioeconomic status of a population is a key and decisive factor in choosing the type of daily fuel consumed, especially in rural areas [44]. Socioeconomic development in low- and middle-income countries may be an important driver of the decline in biomass use [45]. At the same time, people’s socioeconomic status determines their access to social resources and the diversity of their food choices. According to Das et al., as household income increases, the possibility of choosing clean cooking fuels over solid fuels increases [46]. Moreover, Ouedraogo [47] revealed that a household’s desire to consumption clean energy is influenced by educational standing.

There is a link between SES and depression in older adults. A study examining socioeconomic variables and depression in older adults in six low- and middle-income countries suggested that both social and economic factors play an important role in the onset, diagnosis, management and prevention of depression in older adults [48]. Gallo and Matthews’ theoretical framework indicates that having a low SES lowers an individual’s ability to regulate stress, making them more vulnerable to negative feelings and thoughts [49]. It has also been argued that having a higher SES encourages interpersonal ties and social networks, which may help to lessen the occurrence of depressive symptoms [50]. Studies have shown that having a low socioeconomic status increases loneliness [51] and loneliness has been linked to depression [52]. Individuals with higher income were able to avoid hazard factors and adverse exposure [53]. High levels of education may boost human capability and personal capital [54] while also lowering dangerous behaviors and poor lifestyle habits [55, 56], thereby reducing the risk of depression.

### Strengths and limitations

To our knowledge, our research is the first to investigate the role of the mediating effect of solid fuel consumption on the relationship between SES and depression by using a large and nationally representative sample of older people. However, there are also several limitations to our study. First, this study only collected data on fuel consumption type, and did not collect data on the frequency of consumption, blended fuel consumption or furnace use, which could not be assessed. Second, this study focused on older Chinese individuals, and it is prudent that the result be extended to other populations or countries/regions. Third, differences in demographic characteristics, lifestyle behaviors and health status between the missing participants and study participants may have influenced our results. Fourth, the specific type of ventilation system used was not investigated in this study. Traditional Chinese kitchen ventilation systems tend to include a chimney and a smoke ventilator, and the effects of the ventilation system type may be obscured [57]. Fifth, the mediation method used in this study has some limitations and the precision of the test may be relatively low [58]. Sixth, this study did not observe the effect of cooking on depression, and further study is needed to explore this relationship in the future. Finally, the cross-sectional data were used to explore the correlation among socioeconomic status, solid fuel consumption and depression in older adults but not causality in our study. Further cohort studies are needed to examine causality.

## Conclusion

Our findings suggest that the use of solid fuels is significantly associated with greater odds of depression in older Chinese adults, and that SES is also associated with depression. Solid fuel use may mediate the relationship between SES and depression.

### Electronic supplementary material

Below is the link to the electronic supplementary material.

Supplementary Material 1

## Data Availability

Data are from the Chinese Longitudinal Healthy Longevity Survey, which is a public, open access repository (https://opendata.pku.edu.cn).

## Abbreviations

CLHLS: Chinese Longitudinal Healthy Longevity Survey
SES: Socioeconomic status
SD: Standard deviation
OR: Odds ratio
CI: Confidence interval
CES-D-10: The 10-item Center for Epidemiologic Studies Depression Scale
BMI: Body mass index
PM: Particulate matter

## References

[1] Fullerton DG, Bruce N, Gordon SB (2008). Indoor air pollution from biomass fuel smoke is a major health concern in the developing world. Trans R Soc Trop Med Hyg.

[2] GBD 2015 Risk Factors Collaborators (2016). Global, regional, and national comparative risk assessment of 79 behavioural, environmental and occupational, and metabolic risks or clusters of risks, 1990–2015: a systematic analysis for the global burden of Disease Study 2015. Lancet.

[3] Wang Q (2018). Urbanization and Global Health: the role of Air Pollution. Iran J Public Health.

[4] World Health Organization. Household Air Pollution and Health. 2018. https://www.who.int/news-room/fact-sheets/detail/household-air-pollution-and-health. Accessed on 30 Jul, 2023.

[5] Zheng X (2016). Chinese Household Energy Consumption Report 2015.

[6] Zeng Y (2012). Towards deeper Research and Better Policy for Healthy Aging - -using the Unique Data of Chinese Longitudinal Healthy Longevity Survey. China Economic J.

[7] National Bureau of Statistics of China. http://www.stats.gov.cn/english/. Accessed on 30 Jul 2023.

[8] Fang EF, Scheibye-Knudsen M, Jahn HJ, Li J, Ling L, Guo H (2015). A research agenda for aging in China in the 21st century. Ageing Res Rev.

[9] Byrne GJ, Pachana NA (2010). Anxiety and depression in the elderly: do we know any more?. Curr Opin Psychiatry.

[10] Zhong BL, Ruan YF, Xu YM, Chen WC, Liu LF (2020). Prevalence and recognition of depressive disorders among Chinese older adults receiving primary care: a multi-center cross-sectional study. J Affect Disord.

[11] Blazer DG (2003). Depression in late life: review and commentary. J Gerontol A Biol Sci Med Sci.

[12] Li C, Zhou Y, Ding L (2021). Effects of long-term household air pollution exposure from solid fuel use on depression: evidence from national longitudinal surveys from 2011 to 2018. Environ Pollut.

[13] Shao J, Ge T, Liu Y, Zhao Z, Xia Y (2021). Longitudinal associations between household solid fuel use and depression in middle-aged and older Chinese population: a cohort study. Ecotoxicol Environ Saf.

[14] Liu Y, Chen X, Yan Z (2020). Depression in the house: the effects of household air pollution from solid fuel use among the middle-aged and older population in China. Sci Total Environ.

[15] Banerjee M, Siddique S, Dutta A, Mukherjee B, Ranjan Ray M (2012). Cooking with biomass increases the risk of depression in pre-menopausal women in India. Soc Sci Med.

[16] Li Q, Jiang J, Wang S, Rumchev K, Mead-Hunter R, Morawska L, Hao J (2017). Impacts of household coal and biomass combustion on indoor and ambient air quality in China: current status and implication. Sci Total Environ.

[17] MohanKumar SM, Campbell A, Block M, Veronesi B (2008). Particulate matter, oxidative stress and neurotoxicity. Neurotoxicology.

[18] Block ML, Calderón-Garcidueñas L (2009). Air pollution: mechanisms of neuroinflammation and CNS disease. Trends Neurosci.

[19] Calderón-Garcidueñas L, Calderón-Garcidueñas A, Torres-Jardón R, Avila-Ramírez J, Kulesza RJ, Angiulli AD (2015). Air pollution and your brain: what do you need to know right now. Prim Health Care Res Dev.

[20] Hahad O, Lelieveld J, Birklein F, Lieb K, Daiber A, Münzel T (2020). Ambient air Pollution increases the risk of Cerebrovascular and Neuropsychiatric disorders through induction of inflammation and oxidative stress. Int J Mol Sci.

[21] Salem Ali Albar HM, Ali N, Musstjab Akber Shah Eqani SA, Alhakamy NA, Nazar E, Rashid MI (2020). Trace metals in different socioeconomic indoor residential settings, implications for human health via dust exposure. Ecotoxicol Environ Saf.

[22] Ferguson L, Taylor J, Davies M, Shrubsole C, Symonds P, Dimitroulopoulou S (2020). Exposure to indoor air pollution across socio-economic groups in high-income countries: a scoping review of the literature and a modelling methodology. Environ Int.

[23] McCarron A, Uny I, Caes L, Lucas SE, Semple S, Ardrey J, Price H (2020). Solid fuel users’ perceptions of household solid fuel use in low- and middle-income countries: a scoping review. Environ Int.

[24] Matthews KA, Gallo LC (2011). Psychological perspectives on pathways linking socioeconomic status and physical health. Annu Rev Psychol.

[25] Lazzarino AI, Hamer M, Stamatakis E, Steptoe A (2013). Low socioeconomic status and psychological distress as synergistic predictors of mortality from stroke and coronary heart disease. Psychosom Med.

[26] Virtanen M, Kawachi I, Oksanen T, Salo P, Tuisku K, Pulkki-Råback L, Pentti J, Elovainio M, Vahtera J, Kivimäki M (2011). Socio-economic differences in long-term psychiatric work disability: prospective cohort study of onset, recovery and recurrence. Occup Environ Med.

[27] Qiu S, Chen X, Chen X, Luo G, Guo Y, Bian Z (2022). Solid fuel use, socioeconomic indicators and risk of cardiovascular diseases and all-cause mortality: a prospective cohort study in a rural area of Sichuan, China. Int J Epidemiol.

[28] Zeng Y, Feng Q, Hesketh T, Christensen K, Vaupel JW (2017). Survival, disabilities in activities of daily living, and physical and cognitive functioning among the oldest-old in China: a cohort study. Lancet.

[29] Andresen EM, Malmgren JA, Carter WB, Patrick DL (1994). Screening for depression in well older adults: evaluation of a short form of the CES-D (center for epidemiologic studies Depression Scale). Am J Prev Med.

[30] Liu H, Xu X, Hall JJ, Wu X, Zhang M (2016). Differences in depression between unknown diabetes and known diabetes: results from China health and retirement longitudinal study. Int Psychogeriatr.

[31] Cheng ST, Chan AC (2005). The Center for epidemiologic studies Depression Scale in older Chinese: thresholds for long and short forms. Int J Geriatr Psychiatry.

[32] Cheng ST, Chan AC (2008). Detecting depression in Chinese adults with mild dementia: findings with two versions of the Center for epidemiologic studies Depression Scale. Psychiatry Res.

[33] Cole MG, Dendukuri N (2003). Risk factors for depression among elderly community subjects: a systematic review and meta-analysis. Am J Psychiatry.

[34] Lim YH, Kim H, Kim JH, Bae S, Park HY, Hong YC (2012). Air pollution and symptoms of depression in elderly adults. Environ Health Perspect.

[35] Vert C, Sánchez-Benavides G, Martínez D, Gotsens X, Gramunt N, Cirach M (2017). Effect of long-term exposure to air pollution on anxiety and depression in adults: a cross-sectional study. Int J Hyg Environ Health.

[36] Baron RM, Kenny DA. (1986) The moderator-mediator variable distinction in social psychological research: conceptual, strategic, and statistical considerations. *J Pers Soc Psychol* 1986;51:1173–1182. 10.1037//0022-3514.51.6.1173. 10.1037//0022-3514.51.6.1173 3806354

[37] Iacobucci D (2012). Mediation analysis and categorical variables: the final frontier. J Consum Psychol.

[38] Cheng HG, Chen S, McBride O, Phillips MR (2016). Prospective relationship of depressive symptoms, drinking, and tobacco smoking among middle-aged and elderly community-dwelling adults: results from the China Health and Retirement Longitudinal Study (CHARLS). J Affect Disord.

[39] Hasler G (2010). Pathophysiology of depression: do we have any solid evidence of interest to clinicians?. World Psychiatry.

[40] Li H, Cai J, Chen R, Zhao Z, Ying Z, Wang L (2017). Particulate matter exposure and stress hormone levels: a Randomized, Double-Blind, crossover trial of Air Purification. Circulation.

[41] Stetler C, Miller GE (2011). Depression and hypothalamic-pituitary-adrenal activation: a quantitative summary of four decades of research. Psychosom Med.

[42] Rose M, Filiatreault A, Guénette J, Williams A, Thomson EM (2020). Ozone increases plasma kynurenine-tryptophan ratio and impacts hippocampal serotonin receptor and neurotrophic factor expression: role of stress hormones. Environ Res.

[43] Jaworek AK, Jaworek M, Makara-Studzińska M, Szafraniec K, Doniec Z, Szepietowski J (2022). Depression and serum content of serotonin in adult patients with atopic dermatitis. Adv Exp Med Biol.

[44] Zhu L, Liao H, Hou B, Cheng L, Li H (2020). The status of household heating in northern China: a field survey in towns and villages. Environ Sci Pollut Res Int.

[45] Hickman JE, Andela N, Tsigaridis K, Galy-Lacaux C, Ossohou M, Bauer SE. (2021) Reductions in NO2 burden over north equatorial Africa from decline in biomass burning in spite of growing fossil fuel use, 2005 to 2017. *Natl Acad Sci U S A* 2021;118:e2002579118. 10.1073/pnas.2002579118. 10.1073/pnas.2002579118 PMC7896302 33558224

[46] Das S, De Groote H, Behera B (2014). Determinants of household energy use in Bhutan. Energy.

[47] Ouedraogo B (2006). Household energy preferences for cooking in urban Ouagadougou, Burkina Faso. Energy Policy.

[48] Brinda EM, Rajkumar AP, Attermann J, Gerdtham UG, Enemark U, Jacob KS (2016). Health, Social, and Economic Variables Associated with Depression among Older people in Low and Middle Income countries: World Health Organization Study on Global AGEing and Adult Health. Am J Geriatr Psychiatry.

[49] Gallo LC, Matthews KA (2003). Understanding the association between socioeconomic status and physical health: do negative emotions play a role?. Psychol Bull.

[50] Zhang J, Hu H, Hennessy D, Zhao S, Zhang Y (2019). Digital media and depressive symptoms among Chinese adolescents: a cross-sectional study. Heliyon.

[51] Domènech-Abella J, Mundó J, Lara E, Moneta MV, Haro JM, Olaya B (2017). The role of socio-economic status and neighborhood social capital on loneliness among older adults: evidence from the Sant Boi Aging Study. Soc Psychiatry Psychiatr Epidemiol.

[52] Cacioppo JT, Hawkley LC, Thisted RA (2010). Perceived social isolation makes me sad: 5-year cross-lagged analyses of loneliness and depressive symptomatology in the Chicago Health, Aging, and Social relations Study. Psychol Aging.

[53] Pescosolido BA, Martin JK, McLeod JD, Rogers A. Handbook of the sociology of health, illness, and healing: a blueprint for the 21st century. Springer Science & Business Media; 2010.

[54] Montez JK, Hummer RA, Hayward MD (2012). Educational attainment and adult mortality in the United States: a systematic analysis of functional form. Demography.

[55] Brunello G, Fort M, Schneeweis N, Winter-Ebmer R (2016). The Causal Effect of Education on Health: what is the role of Health behaviors?. Health Econ.

[56] Margolis R (2013). Educational differences in healthy behavior changes and adherence among middle-aged americans. J Health Soc Behav.

[57] Carter EM, Shan M, Yang X, Li J, Baumgartner J (2014). Pollutant emissions and energy efficiency of Chinese gasifier cooking stoves and implications for future intervention studies. Environ Sci Technol.

[58] VanderWeele TJ (2016). Mediation analysis: a practitioner’s guide. Annu Rev Public Health.

