# Supplementary material for: Solid fuel use, socioeconomic status and depression: a cross-study of older adults in China
Source: BMC Geriatr. 2024 Jan 30;24:115. doi: 10.1186/s12877-024-04670-6 (PMC10829389; doi:10.1186/s12877-024-04670-6)
Supplement: Supplementary file 1 — Supplementary Material 1 [file 12877_2024_4670_MOESM1_ESM.docx]

**Supplementary material**

**The first part**

Figure S1. Mediation analysis diagram

| $Y=cX+e_{1}$ | (Equation 1) |
| --- | --- |
| $M=aX+e_{2}$ | (Equation 2) |
| $Y=c^{'}X+bM+e_{3}$ | (Equation 3) |
| $Z_{\mathrm{ab}}=\frac{Z_{a\times b}}{\mathrm{SE}\left( Z_{a\times b} \right)}=\frac{Z_{a}\times Z_{b}}{\sqrt{Z_{a}^{2}+Z_{b}^{2}+1}}$ | (Equation 4) |
| $Mediation Effect Percentage=\frac{\mathrm{ab}}{c}\times100\%$ | (Equation 5) |

Table S1 Coefficients of covariates in the mediation effect

|  | CES-D-10(cut-off value = 10) | | | CES-D-12(cut-off value = 12) | | | |
| --- | --- | --- | --- | --- | --- | --- | --- |
|  | Equation 1 | Equation2 | Equation 3 | | Equation 1 | Equation2 | Equation 3 |
| **Education** | | | | | | | |
| Age(years) | -0.01* | -0.02* | -0.01* | | -0.01* | -0.02* | -0.01* |
| Sex | 0.16* | 0.02 | 0.16* | | 0.15* | 0.02 | 0.15* |
| Residence status | -0.16* | 1.71* | -0.21* | | -0.18* | 1.71* | -0.22* |
| Marital status | 0.15* | -0.29* | 0.16* | | 0.16* | -0.29* | 0.18* |
| Smoking status | 0.06 | -0.15 | 0.06 | | 0.07 | -0.15 | 0.07 |
| Drinking status | 0.24* | -0.18* | 0.25* | | 0.34* | -0.18* | 0.34* |
| Ventilation status | -0.43* | -0.74* | -0.40* | | -0.45* | -0.74* | -0.41* |
| Participate in social activities | 0.11 | -0.39* | 0.12 | | 0.23 | -0.39* | 0.24* |
| Play cards status | -0.27* | -0.17* | -0.26* | | -0.19* | -0.17* | -0.18 |
| Exercise status | 0.60* | 0.51* | 0.58* | | 0.69* | 0.51* | 0.68* |
| Tourism status | -0.34* | -0.72* | -0.33* | | -0.32* | -0.72* | -0.30* |
| Fresh fruit | -0.27* | -0.21* | -0.26* | | -0.31* | -0.21* | -0.30* |
| Vegetables | -0.31* | -0.01 | -0.31* | | -0.33* | -0.01 | -0.33* |
| BMI (kg/m2) | -0.10* | -0.17* | -0.09* | | -0.13* | -0.17* | -0.13* |
| Hypertension | -0.05 | 0.08 | -0.06 | | -0.04 | 0.08 | -0.04 |
| Diabetes | 0 | 0.17* | -0.01 | | 0.01 | 0.17* | 0 |
| Heart diseases | -0.08 | 0.14* | -0.09 | | -0.15* | 0.14* | -0.16* |
| Stroke | 0 | -0.02 | 0 | | -0.06 | -0.02 | -0.06 |
| **Annual Household Income** | | | | | | | |
| Age(years) | 0 | -0.01* | 0 | | -0.01 | -0.01* | -0.01 |
| Sex | 0.22* | 0.12 | 0.22* | | 0.19 | 0.12 | 0.19* |
| Residence status | -0.22* | 1.45* | -0.25* | | 0.24* | 1.45* | -0.26* |
| Marital status | 0.16* | -0.25* | 0.17* | | 0.18* | -0.25* | 0.18* |
| Smoking status | 0.08 | -0.06 | 0.08 | | 0.09 | -0.06 | 0.09 |
| Drinking status | 0.24* | -0.18* | 0.24* | | 0.34* | -0.18* | 0.34* |
| Ventilation status | -0.40* | -0.61* | -0.38* | | -0.41* | -0.61* | -0.39* |
| Participate in social activities | 0.11 | -0.38* | 0.11 | | 0.23 | -0.38* | 0.23* |
| Play cards status | -0.27* | -0.14 | -0.26* | | -0.19 | -0.14 | -0.18 |
| Exercise status | 0.60* | 0.52* | 0.59* | | 0.69* | 0.52* | 0.68* |
| Tourism status | -0.32* | -0.60* | -0.31* | | -0.29* | -0.60* | -0.28* |
| Fresh fruit | -0.26* | -0.17* | -0.26* | | -0.30* | -0.17* | -0.30* |
| Vegetables | -0.31* | 0.02 | -0.31* | | -0.33* | 0.02 | -0.33* |
| BMI (kg/m2) | -0.09* | -0.18* | -0.09* | | -0.13* | -0.18* | -0.13* |
| Hypertension | -0.06 | 0.06 | -0.06 | | -0.04 | 0.06 | -0.04 |
| Diabetes | -0.01 | 0.15* | -0.01 | | 0 | 0.15* | 0 |
| Heart diseases | -0.08 | 0.11 | -0.09 | | -0.16* | 0.11 | -0.16* |
| Stroke | -0.01 | -0.03 | -0.01 | | -0.06 | -0.03 | -0.06 |

CES-D, Center for Epidemiological Studies Depression Scale.

**P*＜0.05

**The second part**

The specific process of stepwise technique：

(1) Test the significance of the regression coefficient **c** (Equation 1) of socioeconomic status on depression.

(2) Test the significance of the regression coefficient **a** (Equation 2) of socioeconomic status on solid fuel.

(3) The significance of the regression coefficients **b** and **c'** (Equation 3) of socioeconomic solid fuel and status on depression.
